# Comparing Balance Performance on Force Platform Measures in Individuals with Parkinson's Disease and Healthy Adults

**DOI:** 10.1155/2018/6142579

**Published:** 2018-12-02

**Authors:** Cathy C. Harro, Amanda Kelch, Cora Hargis, Abigail DeWitt

**Affiliations:** ^1^Assistance Professor, Physical Therapy, College of Health Professions, Grand Valley State University, Cook DeVos Center for Health Sciences, 301 Michigan Street NE, Suite 268, Grand Rapids, MI 49503, USA; ^2^Mary Free Bed Rehabilitation Hospital, 235 Wealthy Street SE, Grand Rapids, MI 49503, USA; ^3^PT Solutions, 1321 Tusculum Blvd., Greeneville, TN 37743, USA

## Abstract

**Introduction:**

Postural instability is a known contributing factor to balance dysfunction and increased fall risk in those with Parkinson's disease (PD). Computerized posturography employing a force platform system provides objective, quantitative assessments of postural control impairments. This study examines balance performance as measured by force platform (FP) tests in persons with PD compared to age-matched healthy adults. Secondarily, we examine if these FP measures provide diagnostic and clinically meaningful information about the underlying balance impairments in the PD population.

**Methods:**

Participants—42 individuals with PD (Hoehn and Yahr stage = 2.33 ± 0.77) and 55 age-matched healthy adults—were assessed on three standardized balance measures on a computerized force platform system. Between groups, comparisons of FP performance were analyzed using independent *t*-test. Within the group, comparisons for the PD cohort were analyzed using ANOVA for comparing disease stage and Mann–Whitney *U* test for PD subtypes.

**Results:**

The PD cohort demonstrated significantly greater postural instability on the sensory organization test (SOT) measures (*P*=0.013, CI-95% = 1.286 to 10.37) and slower movement velocity on the limits of stability (LOS) test (*P*=0.001, CI-95% = 0.597 to 1.595) than the healthy cohort, suggesting that these tests were sensitive to detect sensory integration and voluntary postural control deficits in the PD cohort. Within the PD group, the SOT differentiated between H&Y stages 1–3. The motor control test (MCT) detected changes in reactive postural control mainly in later disease stages. All three FP tests distinguished between PD subtypes, with the *Posture Gait Instability* subtype demonstrating poorer balance performance than *Tremor Dominant* subtype.

**Conclusion:**

These findings suggest FP measures provide clinically meaningful, diagnostic information in the examination of balance impairments in individuals with PD. FP measures may inform clinicians regarding intrinsic balance deficits and guide them in designing targeted balance interventions to reduce fall risk in persons with PD.

## 1. Introduction

Parkinson's disease (PD) is a chronic progressive neurological disorder that involves both motor and nonmotor symptoms [1, 2]. The most common motor symptoms of PD include bradykinesia, rigidity, tremor, and postural instability, which result in functional decline and disability [2]. PD is a highly prevalent disorder, as it is estimated that 819,000 individuals will be diagnosed by 2020 and 1.06 million by 2030 [3]. In 2010, it was estimated that those living with PD incurred over 14 billion dollars in medical expenses [3]. It is well established that those with PD are at a high fall risk [4–10]. Research suggests that 35–70% of those with PD experience multiple falls and are at a nine times higher risk for falls than age-matched healthy individuals [4, 5]. Falls can not only lead to serious injury but may also have a negative impact on mobility, activities of daily living, emotional well-being, and quality of life [6]. Therefore, it is important to identify sensitive clinical measures that detect balance impairments to better direct individualized fall prevention programs.

Falls in PD are largely multifactorial and intrinsic in nature [4–6]. Deficits in postural control mechanisms are a key contributor to instability. Those with PD have increased postural sway during both static and dynamic activities, resulting in reduced limits of stability and functional balance [10, 11]. Anticipatory postural strategies are often diminished, possibly due to impairments with feed-forward mechanisms for movement and balance [12]. Individuals with PD also have reduced reactive postural responses, which can result in insufficient balance strategies in response to perturbations and increased fall risk [13]. Additionally, those with PD tend to select inappropriate postural strategies and have abnormal sequencing of muscle activation, leading to inefficient postural strategies [14]. Impaired central processing of proprioceptive and vestibular information also contributes to postural instability, resulting in increased reliance on vision to maintain balance [15, 16].

When examining patients with PD, it is important to assess the mechanisms of postural instability and underlying balance impairments to identify fall risk and guide physical therapy interventions. The Parkinson Evidence Database to Guide Effectiveness and the Movement Disorder Society (MDS) developed recommendations for balance examination in the PD population [17, 18]. However, the recommended balance measures are limited as the majority of these clinical tests evaluate balance at the activity or functional task level without examining the underlying postural control impairments at the body structure and function level [19–21]. King et al. [21] found that functional measures used to assess balance at the activity level were not responsive for detecting changes after balance interventions in persons with PD. In addition, these tests rely primarily on subjective scoring scales [22]. The MDS (2016) suggests that more research is needed on balance and posture control measures with regard to the diagnosis of balance impairments, sensitivity to measure change, and applicability across the disease spectrum [17]. Force platform systems provide objective and quantitative assessments for diagnosis of postural control impairments in persons with PD.

The NeuroCom force platform system is a computerized system used to examine underlying balance deficits in patients with neurologic conditions and in the elderly at fall risk. Standardized force platform (FP) measures on this system include limits of stability (LOS) test, sensory organization test (SOT), and motor control test (MCT), which assess voluntary postural control, sensory integration, and reactive postural control, respectively. These FP measures provide quantitative data on postural sway, movement amplitude and velocity, sensory strategies, and latency of postural responses. These measures have been applied in elderly to identify balance impairments and fall risk. The SOT and LOS test validly identify postural control impairments in the elderly fallers, differentiate fallers from nonfallers, and predict fall risk [23–26]. These FP measures are also supported as reliable and valid measures of underlying postural control deficits across neurological populations [27–30]. Specific to the PD population, Harro et al. [31] reported excellent test-retest reliability for these FP measures, and fair to moderate correlation between the SOT and LOS test and clinical balance measures.

Regarding the PD population, limited research supports that FP measures may identify balance impairments, even in early disease stages, and may detect balance decline with disease progression [32–35]. Rossi-Izquierdo et al. [36] found that the SOT and LOS test differentiated between PD and healthy groups and between fallers from nonfallers within the PD cohort. Colnat-Coulbois et al. [37] reported that the SOT detected postural control deficits in persons in late stage PD. The LOS test identified impaired voluntary postural control in both medial-lateral and posterior directions in persons in the early stages of PD [38, 39]. Reduced movement velocity on the LOS test has been correlated with reduced gait speed and stride length in persons with PD [40]. Frenklach et al. [41] found that postural sway in both static and dynamic conditions on FP significantly increases with PD progression. There is limited evidence specifically regarding the MCT in the PD population, but several foundational studies utilized FP translations to assess reactive postural control strategies and found that persons with PD demonstrated impaired reactive strategies [42–44]. Particularly, in response to posterior perturbations, individuals with PD had smaller stability margins, increased motor response latency, and took more steps than healthy controls [42–44]. This preliminary evidence suggests that FP measures may provide important diagnostic information regarding balance function and decline with disease progression in PD, but further research is needed. There is also limited research supporting the use of FP measures to measure changes in postural control following exercise-based interventions in PD [45, 46].

Further research on the clinical utility of the FP measures in the PD population is warranted due to the current gaps in the literature. Although there are valid and reliable clinical measures of balance at the functional level, there is a lack of rigorous research on balance measures at the impairment level. Further research is needed on computerized FP measures to assess if these measures identify balance deficits in PD across the disease stages and are able to sensitively assess changes in balance function with disease progression. In addition, further research is needed to examine if these measures are able to distinguish between balance impairments in persons with PD and age-related changes in balance in healthy older adults.

The purpose of this study is to compare balance performance on the LOS test, SOT, and MCT in persons with PD with age- and gender-matched healthy adults. Secondarily, this study will assist clinicians in determining if these standardized FP measures provide diagnostic and clinically meaningful information about the underlying balance impairments in the PD population. This research may help clinicians select examination measures to identify balance deficits across disease stages in persons with PD, which may direct balance interventions to reduce fall risk.

## 2. Methods

### 2.1. Research Design

This study examines the differences in postural control between individuals with PD and an age- and gender-matched healthy cohort, as measured by standardized tests on the NeuroCom force platform system. The study analyzes between-group comparisons on the LOS test, MCT, and SOT, and examines if there were differences in test performance within the PD cohort based on disease stage, PD subtype, and age grouping.

### 2.2. Participants

This study's recruitment and participant enrollment took place from June 2014 to March 2016. Recruitment methods included posting information in local Parkinson association newsletters, posting flyers in the community and local university, and conducting informational meetings at local PD support groups and community exercise classes. Recruitment of age- and gender-matched healthy adult controls was based on the following age groupings: 41–50, 51–60, 61–70, and 71–80 years old. Inclusion and exclusion criteria used for both cohorts are listed in Table 1.

Researchers conducted phone interviews to determine if participants met the inclusion and exclusion criteria, followed by an in-person visit to assess walking and stair ability and to administer the Montreal Cognitive Assessment (MoCA) and the Semmes Weinstein monofilament (SWME). A MoCA cutoff score of 21/30 points for identifying dementia was used for both groups (sensitivity 81%, specificity 95% in PD) [47]. SWME scores of five or more insensate points (out of 8 testing points) were considered indicative of sensory neuropathy (sensitivity of 0.77, specificity of 0.96) [48]. Those individuals who met the inclusion criteria were enrolled in the study and completed the informed consent process. The Grand Valley State University Institutional Review Board approved this study.

Medical history, activity self-report, and history of falls within the last six months were collected from all participants and/or their spouses. Falls were defined as any instance in which the individual lost their balance, causing them to fall to the ground or hit an object below them. Participants were classified as “fallers” if they reported 2 or more falls in the last 6 months. Information regarding disease characteristics and severity for the PD cohort was gathered through the freezing of gait questionnaire (FOGQ) and the Movement Disorder Society-sponsored revision of the unified Parkinson's disease rating scale (MDS-UPDRS), which was administered by the principal investigator who had completed training and certification by the MDS. Participants were classified as “freezers” if they scored greater than one on the third item of the FOGQ. PD participants were classified into three PD subtypes based on their MDS-UPDRS score as described by Stebbins et al.: (1) tremor predominant (TD), (2) postural instability and gait difficulty (PIGD), and (3) indeterminate (I) [49].

**Figure 1:**
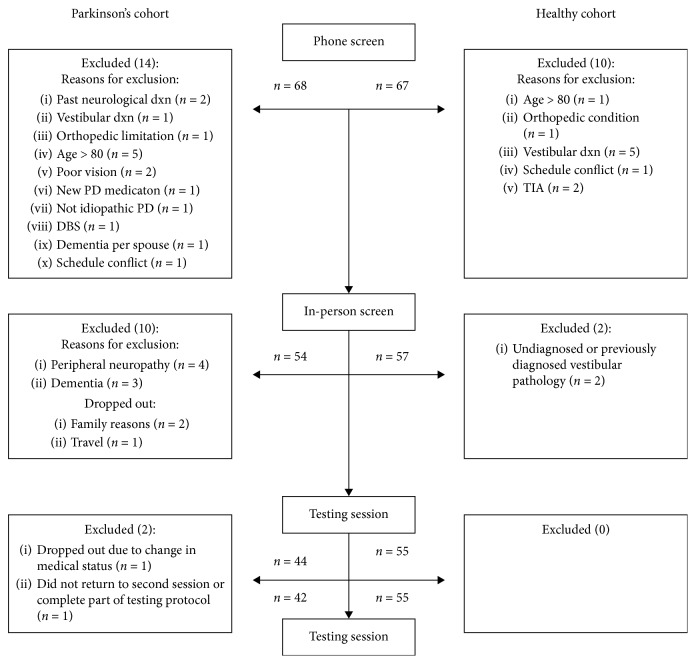
Participant flow diagram.

As depicted in Figure 1, 68 individuals for the PD cohort and 67 individuals for the healthy cohort were recruited, with 42 and 55 qualified participants in the respective cohorts.  Table 2 provides summary of group demographics. The mean Hoehn and Yahr (H&Y) stage in the PD cohort was 2.33 (±0.77), and the mean disease duration was 53.90 (±37.86) months. In the PD cohort, 24% were “fallers,” and 24% were classified as “freezers.” There were no “fallers” in the healthy cohort.

### 2.3. Testing Procedures

**Figure 2:**
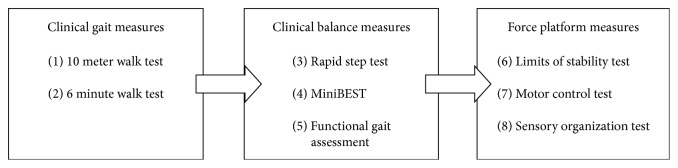
Testing protocol utilized for both Parkinson's disease and healthy cohorts.

This study was part of a larger study examining the reliability and concurrent validity of FP measures in individuals with PD, with detailed description of the full study's methods published by Harro et al. [31]. Data were collected in the university's biomechanics laboratory, a quiet, controlled environment. Participants completed a 1.5-hour testing session as outlined in Figure 2, with rest periods provided between testing segments. Tests were conducted in a standardized order for both cohorts. A second test session was completed within 10 days of the first session with readministration of the FP tests and rapid step test (RST) in order to examine test-retest reliability of these measures. This reliability data is published in a separate paper [31]. The PD cohort was tested during the “on time” of their medication and at a similar time of day for both test sessions. Each group of tests was carried out by the same researcher to maintain consistency and standardize the testing procedures.

### 2.4. Force Platform Measures

The NeuroCom™ Smart Equitest Clinical Research System/Balance Master System 9.1 was used to administer the LOS test, MCT, and SOT (Natus Medical Inc., 9570 SE Lawnfield Rd, Clackamas, OR, 97015). Participants were barefoot for the FP testing. Foot placement on the platform was carefully controlled, and standardized instructions were given for each test. Per testing protocol guidelines, participants wore an overhead harness and were closely guarded during testing. A “fall” during any of the tests was defined as needing to take a step, using hands on the walls, or requiring assistance from the researcher to regain balance. Reliability and validity of these measures in persons with PD was discussed earlier in this paper [27–29, 31].

#### 2.4.1. Limits of Stability (LOS)

This test assesses voluntary postural control as demonstrated by participants moving their center of gravity towards eight targets when cued by a visual signal on-screen. The test variables recorded for this study were endpoint excursion (EPE), average movement velocity (Avg MV), endpoint excursion to posterior targets 4, 5, and 6 (EPE 4–6), total number of falls, and number of falls during posterior targets 4, 5, and 6.

#### 2.4.2. Motor Control Test (MCT)

This test assesses reactive postural control in response to anterior and posterior perturbations induced by movements of the force platform surface. The test variables recorded for this study were the average response latency (Avg Lat), average amplitude of response to large perturbations (Avg Amp L), average posterior amplitude (Avg Posterior Amp), and average posterior latency (Avg Posterior Lat) in response to posterior perturbations.

#### 2.4.3. Sensory Organization Test (SOT)

This test measures postural sway in response to six different sensory conditions that challenge the integration of vision, somatosensory, and vestibular senses. The test variables recorded in this study were composite equilibrium score (Comp Eq), vestibular ratio (Vestib Ratio), visual preference ratio, total number of falls, and number of falls in conditions 5 and 6.

### 2.5. Data Analysis

Data collected were used to examine if there were differences in performance on the FP tests between the PD cohort and the age-matched healthy cohort. Using multiple linear regression, it was determined that 46 participants were needed in the PD cohort to maintain a statistical power of 0.80 and achieve an effect size of 0.35 and an alpha level of 0.05 [50]. Therefore, we were slightly underpowered (*N*=42). Descriptive statistics were analyzed to determine the mean and standard deviation for the primary FP variables (SOT Comp Eq, LOS Avg EPE, and MCT Avg Lat) and secondary FP variables including (1) LOS test measures (Avg MV, EPE 4–6, total number of falls, and number of posterior falls 4–6), (2) MCT measures (Avg Amp L, Avg Posterior Amp, and Avg Posterior Lat), and (3) SOT measures (total number of falls, number of falls in conditions 5 and 6, Vestib Ratio, and visual preference).

Discriminative validity was analyzed between the PD and healthy cohorts by using unpooled independent *t*-tests for all FP variables, alpha level set at *P* < 0.05. ANCOVA analysis was utilized to examine whether performance on the primary FP measures varied between the cohorts when controlling for age as a covariate. Estimated variance was reported for variables in which significant between-group differences were found [51].

The secondary research purpose was addressed using subgroup analysis within the PD cohort to examine if FP performance differed based on disease severity, age grouping, and PD subtype (TD and PIGD). Comparison of H&Y stage 4 was excluded as there was only one participant in this group. One-way ANOVA was utilized to assess if there were differences in mean performance on the LOS Avg EPE and Avg MV and MCT Avg Lat among the disease stages. Post hoc analysis was completed with adjusted Bonferroni corrections for multiple comparisons. Kruskal–Wallis analysis was used to assess if there were differences in median performance on the SOT Comp Eq among disease stages, as equality of variance could not be assumed. Post hoc analysis was completed using a Wilcoxon rank-sum test with adjusted Bonferroni correction for multiple comparisons (*P* < 0.017).

Subgroup analyses were performed for the PD cohort based on the PD subtype (PIGD and TD). Subgroup analyses for the primary FP variables (SOT Comp Eq, LOS Avg EPE, and MCT Avg Lat) are reported by Harro et al. in a previous paper [31]. The Mann–Whitney *U* test was analyzed to determine if there were differences between PD subtypes, TD and PIGD, for the secondary FP variables.

A one-way ANOVA was used to assess if there were differences in mean performance on primary FP variables based on the age group within the PD cohort. Age groups were established as group 1 (41–60 years), group 2 (61–70 years), and group 3 (71–80 years) [52]. Post hoc analysis was completed with adjusted Bonferroni corrections for multiple comparisons.

## 3. Results

### 3.1. Between-Group Comparisons on FP Measures

**Figure 3:**
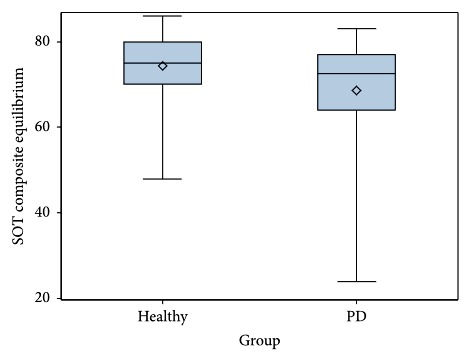
Comparative boxplot of sensory organization test composite equilibrium score for Parkinson's disease and age-matched healthy cohorts.

**Figure 4:**
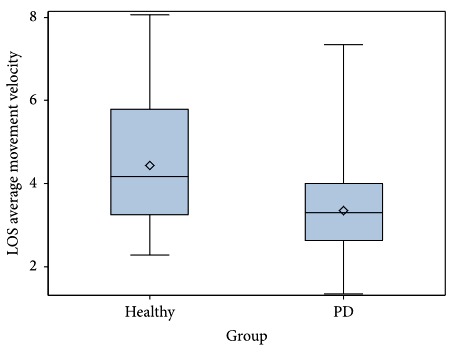
Comparative boxplot of limits of stability average movement velocity performance for Parkinson's disease and age-matched healthy cohorts. LOS, limits of stability.

Descriptive statistics for FP balance measures for both the PD and healthy cohorts are summarized in Table 3. Significant differences between the PD and healthy cohorts in mean performance were found for the following SOT variables: composite equilibrium (*P*=0.013, CI-95% = 1.286 to 10.37), vestibular ratio (*P*=0.027, CI-95% = 0.12 to 0.185), and number of total falls (*P*=0.015, CI-95% = −1.527 to −0.175) (Table 4). Individuals with PD demonstrated significantly lower SOT composite equilibrium scores (PD: *M* = 68.52 (SD 12.93) vs. healthy: *M* = 74.35 (SD 8.05), Figure 3) and vestibular ratio scores (PD: *M* = 0.54 (SD 0.24) vs. healthy: *M* = 0.64 (SD 0.16)). The PD cohort had significantly more falls on the SOT (PD: *M* = 1.21 (SD 2.07) vs. healthy: *M* = 0.36 (SD 0.80)). No significant differences were found between the cohorts in performance on the LOS test or MCT, with the exception of LOS average movement velocity (*P*=0.001, CI-95% = 0.597 to 1.595, Figure 4). The PD had a lower mean average movement velocity than the healthy cohort (PD: *M* = 3.35 (SD 1.02) m/s vs. healthy: *M* = 4.45 (SD 1.45) m/s). There was high variability in the PD cohort for both the SOT Comp Eq and LOS Ave MV measures (Figures 3 and 4).

ANCOVA analysis examined whether FP performance varied between the PD and healthy cohorts when controlling for age. The overall models for all three primary variables were significant (SOT Comp Eq *F*(2,94) = 7.95, *P*=0.001; LOS Avg EPE *F*(2,94) = 16.41, *P* < 0.0001; MCT Avg Lat *F*(2,94) = 4.28 (2), *P*=0.017)). However, SOT Comp Eq was the only variable in which significant between-group differences were found when controlling for age (*F*(2,9) = 6.4, *P*=0.013). Based on estimates from this analysis, a participant in the healthy cohort had, on average, a SOT Comp Eq score of 5.28 points higher than a participant of the same age in the PD cohort.

### 3.2. Comparison of PD Stage, Subtype, and Age Group

**Figure 5:**
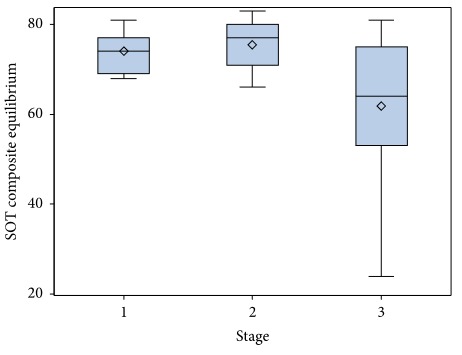
Comparative boxplot of sensory organization test composite equilibrium performance of Parkinson's disease cohort based on the Hoehn and Yahr stage. Hoehn and Yahr stage 4 was not included in this analysis as only one participant was represented in this stage. SOT, sensory organization test.

Within the PD cohort, significant differences were found in MCT Avg Lat among H&Y stages (*F*(2,38) = 4.20, *P*=0.023). Post hoc analysis demonstrated significant differences between H&Y stages 1 and 3 (*P*=0.028), as individuals in stage 3 had slower postural latencies than those in stage 1 (*M* = 147.74 (SD 7.30) m/s; vs. *M* = 137.29 (SD 7.23); respectively). There were also significant differences in median SOT Comp Eq among H&Y stages (*x* ^2^(2) = 10.949, *P*=0.004). Post hoc analysis revealed that there was a significant difference in the median SOT Comp Eq score between those in H&Y stages 2 and 3 (*P*=0.001) and those in stages 1 and 3 (*P*=0.017)( Figure 5). Individuals in stage 3 (Mdn = 61.79) had lower postural stability scores on SOT than those in stages 1 or 2 (Mdn = 74.00; Mdn = 75.47; respectively). No significant differences were found among disease stages for the LOS test variables, Avg EPE (*F* = (2,38) 2.68, *P*=0.082) or Avg MV (*F*(2,38) = 0.41, *P*=0.665).

**Figure 6:**
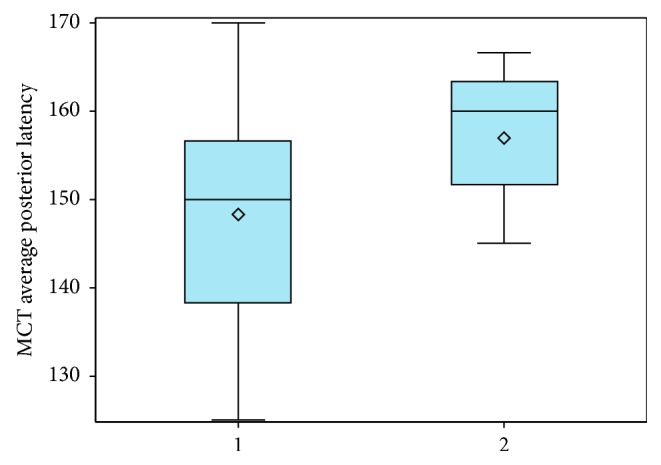
Comparative boxplot of motor control test posterior latency performance of Parkinson's disease cohort based on the PD subtype. Subtype 3 was not included in this analysis as it is an intermediate group with symptoms specific to both subtypes 1 and 2. MCT, motor control test. 1: tremor dominant. 2: postural instability and gait dysfunction.

Previous analysis of the primary FP measures in this study, Harro et al. [31], revealed significant differences between PD subtypes in SOT Comp Eq, LOS EPE, and MCT Ave Lat, with the PIGD group performing poorer on all primary FP balance measures than the TD group. Further analysis of secondary FP measures revealed significant differences between PD subtypes, TD and PIGD, for median performance in LOS EPE 4, 5, and 6 (*Z* = 1.89, *P*=0.029) and MCT Avg Post Lat (*Z* = −2.19, *P*=0.009) (Figure 6). The voluntary LOS excursion to posterior targets was less in the PIGD group (Mdn = 55.00%) compared to the TD group (Mdn = 71.00%), and median reactive latency to posterior perturbations was longer in the PIGD group (Mdn = 160 ms) than the TD group (Mdn = 155 ms). Additionally, the difference in the median number of falls for the SOT was approaching significance (*Z* = −1.617, *P*=0.055), with the PIGD group experiencing a higher number of median falls than the TD group (Mdn = 1, Mdn = 0, respectively).

Significant differences were found between age groups within the PD cohort in LOS Avg EPE (*F*(2,39) = 8.84, *P*=0.01), specifically between the 71- to 80-year-old group and the 41- to 60-year-old group (*M* = 57.88 (SD 13.56) and *M* = 79.73 (SD 13.56), respectively, *P*=0.001) and between the 71–80-year-old group and the 61–70-year-old group (*M* = 57.88 (SD 13.55) and *M* = 72.81 (SD 10.12), *P*=0.005). Limits of stability excursion was significantly reduced in the 71 to 80-year-old age group as compared to the 41–60 and 61–70-year-old age groups. No significant differences were found among age groups for MCT Avg Lat (*F*(2,39) = 3.05, *P*=0.059) and SOT Comp Eq (*F*(2,39) = 1.97, *P*=0.153).

## 4. Discussion

### 4.1. Comparison of Force Platform Measures in PD and Healthy Individuals

This study compared FP measures of balance impairment in persons with PD and an age-matched healthy cohort and demonstrated that sensory organization test (SOT) measures and limits of stability (LOS) movement velocity were significantly different between the two groups. The PD cohort had slower movement velocity during voluntary postural control in the LOS test as compared to healthy controls. Individuals with PD had reduced postural stability on the SOT (Comp Eq score), lower equilibrium scores in conditions requiring effective use of vestibular cues (Vestib ratio), and an increased number of falls during the SOT when compared to healthy controls, reflective of sensory integration deficits affecting balance control. The SOT differentiated between PD and healthy controls, as the Comp Eq score was estimated to be 5.88 higher in healthy individuals than those with PD when controlling for age.

Our findings regarding identification of postural instability based on SOT results in persons with PD compared to healthy individuals are consistent with previous research [7, 37, 41]. Colnat-Coulbois et al. [37] and Rossi et al. [7] also reported that persons with PD had a lower SOT vestibular ratio, reflecting impaired use of vestibular information for balance under varied environmental conditions. Even in early-to-middle disease stages, our findings suggest that persons with PD have postural instability when in challenging or conflicting sensory conditions necessitating the use of the vestibular system. Early identification of sensory processing and integration deficits related to balance function can inform clinicians regarding the need for targeted balance interventions to train the vestibular system to more effectively process and weigh sensory cues, thereby potentially reducing an individual's fall risk.

Individuals with PD displayed reduced movement velocity on the LOS test, which may reflect underlying bradykinesia affecting voluntary movements and impaired feed-forward balance strategies and is consistent with two other studies [7, 40]. Slower, less efficient control of center of mass movements may adversely affect balance during self-generated movements for daily functional tasks, such as picking shoes up off the floor or reaching into a high cabinet. Given that individuals with PD tend to have anteriorly biased postural alignment and often demonstrate difficulty shifting their weight backwards, researchers expected that the LOS endpoint excursion to posterior targets would be significantly reduced compared to healthy controls. Surprisingly, this finding was not supported in our results. However, researchers directly observed that participants with PD used inefficient strategies and were less successful in reaching posterior targets compared to forward and lateral targets during the LOS testing. The majority of both our PD and healthy cohorts were elderly (>60 years old). It is possible that healthy elderly also have an anterior bias in COM, which may have contributed to the lack of significant differences in LOS to posterior targets between the two groups. Previous research reported reduced composite endpoint excursion (EPE) on the LOS test in persons with PD, but this was not supported in our findings [7, 39, 53]. These differences in research findings may be explained by the previous studies' use of different computerized posturography systems and their inclusion of individuals with more advanced stages of PD than our PD cohort.

Currently, there is limited published research on MCT FP performance, a measure of reactive postural control, in persons with early-to-middle stage PD. Dimitrova et al. and Horak et al. [42, 43] examined electromyography during automatic postural responses and reported that individuals with PD had slowed motor latency, inappropriate muscle coactivation, and disordered muscular responses to external perturbations. Our findings from the MCT measures, however, did not show impaired reactive postural control in persons with early-to-middle stage PD. In agreement with our results, Lee et al. did not find any significant differences between healthy and PD cohorts on the MCT [44]. In Horak's study, there was a larger representation of later disease severity (H&Y stages 3 and 4) as compared to our study and Lee's study, which lends support that reactive balance strategies may decline more notably in later stages of PD. Additionally, through partial correlation analysis of this study's data, Harro et al. [31] found that the strongest disease characteristic associated with MCT average latency measure was disease severity, as measured by the UPDRS total score. This finding may further support that MCT is clinically indicated to detect impaired postural responses to perturbations in those in H&Y stages 3 and 4 but not in early stages.

### 4.2. Force Platform Performance of PD Cohort by Disease Severity, PD Subtype, and Age

When examining performance differences within the PD cohort among H&Y stages, the SOT distinguished between those individuals in stages 1 and 3 and those in stages 2 and 3. Additionally, the SOT showed a moderate relationship to disease severity (MDS-UPDRS) [31]. These findings suggest that the SOT may be a good measure to detect decline in sensory processing and integration across disease stages. The SOT provides quantitative data not available from most clinical balance measures and, based on our findings, has particularly good diagnostic value for persons in H&Y stage 3. Likewise, the MCT may sensitively identify a decline in balance with advancing disease, as MCT latency was significantly slower for those in stage 3 as compared to stage 1. These differences in SOT and MCT measures across stages must be interpreted cautiously however, since sample sizes for H&Y stages in this study were small (stage 1 = 7, stage 2 = 15, and stage 3 = 19). Further research should examine this comparison of FP measures across stages with larger subgroup sizes. Both measures may be a valuable addition to the evaluation of postural control in middle stages of PD to assess underlying balance impairments and diagnose if there is a decline in balance function. These measures may also be useful for assessing the effectiveness of directed balance interventions.

Our research findings support that individuals with the posture instability gait Difficulty (PIGD) subtype of PD have greater underlying balance impairments than those with the tremor dominant (TD) subtype. Based on analysis of this PD cohort's performance on primary FP measures, Harro et al. [31] reported that participants in the PIGD subtype had significantly lower SOT Comp Eq scores, lower LOS Avg EPE scores, and slowed MCT average response latencies compared to the TD subtype. Further analysis of secondary FP measures also revealed that the PIGD group had decreased excursion to posterior targets in the LOS test and slowed response latency to posterior perturbations on the MCT compared to the TD group. Regardless of disease stage, subjects in the PIGD subtype demonstrated poorer balance performance on FPM than the TD group, which lends validity for these subgroup classifications. Clinicians may therefore consider the need for more sensitive and diagnostic balance measures in the PIGD subgroup, such as use of FP measures, to identify balance impairments and potential fall risk. This study's analysis of PD subtype was based on 17 subjects in each of the PIGD and TD subgroups. Further research is needed to expand on this analysis with larger sample sizes.

Comparing FP balance performance between age groups within the PD cohort, the LOS Avg EPE was the only variable that demonstrated significant differences. Endpoint excursion to targets was significantly reduced in the 71–80 year-old group when compared to both younger age groups. Since LOS Avg EPE showed no differences between H&Y stages, these results may indicate that age is a greater contributing factor to reduced endpoint excursion than disease stage. Older age (>70 years) combined with advancing PD stage may be a cause for concern of a decline in feedforward, voluntary postural control needed for daily functional tasks.

### 4.3. Clinical Implications

Force platform measures sensitively detect balance impairments in persons with PD and may be especially valuable for individuals with suspected balance underlying impairments based on clinical functional balance measures. The SOT provides clinically valuable information regarding sensory organization strategies for postural control and identifies a decline in these strategies with increasing disease severity in PD. In contrast to the modified clinical test of sensory interaction and balance (Mod-CTSIB), which has fair reliability and validity at best for measuring sensory organization deficits, the SOT has excellent reliability (ICC = 0.90) and good validity [31] and provides quantitative data regarding the use of visual, somatosensory, and vestibular systems for balance under varied environmental conditions. By analyzing a patient's SOT findings, a clinician can create targeted interventions that include multisensory balance training to promote optimal balance function in varied sensory environments. Research supports that multisensory balance training is effective in reducing fall risk and improving balance in persons after stroke and elderly fallers [54, 55]. In individuals with early-to-middle stage PD, multisensory balance training paired with a highly challenging exercise program was shown effective for improving balance and reducing fall rate and fear of falling [56].

The LOS test assesses voluntary postural control and provides quantitative information about limits of stability and speed of center of mass (COM) movement. This study's findings regarding reduced movement velocity on the LOS test in the PD cohort may indicate a need for increased attention in dynamic balance training to address full body bradykinesia. Interventions to improve balance should emphasize full body, self-generated, dynamic movements targeting feed-forward anticipatory strategies for control of balance. Based on our findings, the clinical use of the LOS test may be especially beneficial for those with PD who are categorized in the PIGD subtype and for those in middle to later disease stages to detect balance deficits in feedforward balance mechanisms [7, 40].

Results from this study indicated that there were not deficits in reactive postural control strategies based on MCT results in those in early stages (H&Y 1 and 2). Our findings did support that the MCT appears to be an appropriate diagnostic test to identify reactive balance control deficits for patients in H&Y stages 3 and 4 and in those individuals with PIGD subtype who are at greater risk for early balance decline. If clinical balance assessments indicate suspected deficits in reactive balance strategies, then the MCT may be a useful next step to provide objective, quantitative assessment of this impairment and to assess the efficacy of reactive balance training interventions.

### 4.4. Limitations and Future Research

Limitations in this study related to sample characteristics include a lack of representation of H&Y stage 4 (*N*=1) and the exclusion of individuals with dementia. Therefore, our results regarding the diagnostic value of FP measures cannot be generalized to later disease stages or to those persons with significant cognitive deficits. On average, the participants in our PD cohort reported engaging regularly in physical activity. These participants may have been more physically active than the overall PD population, potentially limiting the generalizability to more sedentary persons with PD. It is also necessary to consider the potential for participation bias in both the PD and healthy cohorts. Due to a sample of convenience and the voluntary participation of subjects, it is possible that individuals who were concerned about their balance were more likely to participate in this study. The samples sizes were relatively small for PD subgroup analysis (by stage and PD subtype), yet despite this limitation, significant differences in FP performance were found. Further research is needed with a more robust subgroup sample size to further investigate if there are difference in FP measures across disease severity or subtypes. Finally, as mentioned above, this study was part of a larger study on reliability and validity of FP measures in PD, which involved a 1.5-hour testing protocol of balance and gait measures, with the FP measures taken during the third segment of the testing session. Therefore, fatigue may have played a factor in FP performance for both cohorts. Rest periods in sitting with hydration were provided following each of the 3 testing segments to minimize this factor. No participants in the PD group requested or had to stop the testing session due to fatigue.

Future research regarding the use of FP measures to evaluate balance impairments and detect balance decline in individuals with PD is warranted. This study was representative of individuals in H&Y stages 1–3; therefore, more research is needed to assess the diagnostic value of these FP measures in later disease stages when balance declines more progressively, specifically stage 4. The PD cohort in our study was not a representative sample of those with freezing of gait characteristics and those with positive fall history (24% of our sample had FOG; 24% of sample were fallers). Further research examining the sensitivity of FP measures to identify balance impairments and assess fall risk in individuals with FOG deficits and positive fall history is warranted. It was not the purpose of this study to examine if FP measures were a valid assessment for fall risk and could predict future falls. Research specifically targeting these important clinical questions is needed. In addition, further research examining the utilization of the FP measures to track changes in postural control longitudinally in persons with PD is recommended to shed light on the temporal course of postural control decline in PD. Lastly, computerized posturography assesses postural control mechanisms in the standing position on a force plate system. Research is needed to evaluate the control of dynamic balance during walking tasks in PD, such as varied speeds, during turns, and walking over obstacles, using a motion analysis system. This research would provide valuable insight into postural control deficits that contribute to fall risk during mobility and walking activities in those with PD.

## 5. Conclusions

This study provides evidence for the discriminative validity of force platform measures in assessing postural control impairments in individuals with PD compared to a healthy, age-matched cohort. The sensory organization test measures and the limits of stability test movement velocity measure were able to differentiate between the two cohorts, supporting the premise that these measures detect PD-specific balance impairments related to sensory integration and voluntary postural control, respectively. The PD cohort demonstrated significantly greater postural instability on sensory organization test measures and slower movement velocity on the limits of stability test than the healthy cohort. Additionally, this study's results support the diagnostic value of FP measures to examine underlying balance impairments related to advancing disease severity, PD subtype (PIGD), and older age in individuals with PD. The sensory organization test differentiated between Hoehn and Yahr stages, sensitively detecting postural instability and sensory integration deficits with disease progression. The motor control test also differentiated between disease stages and PD subtypes, with poorer performance found in later disease stages and in those with the postural instability and gait difficulty subtype. The limits of stability test differentiated between PD subtypes and age groups, with poorer balance performance found in those greater than 70 years old and those with PIGD subtype. These findings support that force platform measures may provide clinically meaningful, quantitative information in the examination of balance impairments in individuals with PD. Given the high fall rate and devastating sequelae of falls in individuals with PD, force platform measures may inform clinicians regarding an individual's underlying balance deficits and direct targeted balance interventions to remediate postural control impairments and reduce fall risk.

## Figures and Tables

**Table 1:** Inclusion and exclusion criteria for Parkinson's disease cohort and age-matched healthy cohort.

Participant cohort	Inclusion criteria	Exclusion criteria
Parkinson's disease cohort	(1) Idiopathic PD (2) Dementia based on scores from the Montreal Cognitive Assessment (MoCA) (3) Self-reported vestibular pathology (4) Peripheral neuropathy as assessed by the Semmes Weinstein monofilament test (SWME) (5) Deep brain stimulation (6) Acute orthopedic injury or surgery that limits the ability to walk or ascend and descend stairs within the past three months (7) Inability to speak/understand English	(1) Other neurological diagnosis (2) Hoehn and Yahr stage I–IV (3) Between 20 and 80 years of age (4) Functional vision with or without corrective lenses (5) Ability to walk 300 feet with or without an assistive device with no more than close guard assist (6) Ability to ascend and descend 6 stairs with or without a railing or assistive device with no more than close guard assist (7) Stable PD medication for the last three months per patient report
Healthy age-matched cohort	(1) Between 20 and 80 years of age (2) Functional vision with or without corrective lenses (3) Able to ascend and descend 6 steps without help from another person, with or without railing (4) Able to walk 300 feet without assistance		

**Table 2:** Demographics for Parkinson's disease cohort and age-matched healthy cohort.

	PD cohort	Healthy cohort
Number of participants	*N*=42	*N*=55
Age	66.21 ± 7.92	64.75 ± 8.50
Gender		
Male	*N*=22	*N*=24
Female	*N*=20	*N*=31
Disease duration (months)	53.90 ± 37.86	N/A
Hoehn and Yahr stage	2.33 ± 0.77	
Stage 1	*N*=7	N/A
Stage 2	*N*=15	
Stage 3	*N*=19	
Stage 4	*N*=1	
MDS-UPDRS total score	47.98 ± 23.70	N/A
MDS-UPDRS motor score	25.95 ± 14.51	N/A
Number of medications	4.30 ± 3.01	2.31 ± 1.69
Percentage of fallers	24%	0%
Percentage of freezers	24%	N/A
PD subtype		
(1) Tremor dominant	*N*=17	N/A
(2) Posture instability and gait difficulty	*N*=17	N/A
(3) Indeterminate	*N*=8	N/A

**Table 3:** Descriptive statistics of force platform measures.

Cohort	FP measures	Mean	SD	Minimum	Q1	Median	Q3	Maximum
Parkinson's disease cohort (*N*=42)	SOT composite equilibrium (1–100)	68.52	12.93	24.00	64.00	72.50	77.00	83.00
	SOT vestibular ratio (0-1)	0.54	0.24	0.00	0.47	0.61	0.72	0.85
	SOT visual preference	0.98	0.13	0.69	0.91	0.98	1.04	1.32
	SOT fall number	1.21	2.07	0.00	0.00	0.00	1.00	8.00
	SOT fall number-conditions 5 and 6	1.12	1.82	0.00	0.00	0.00	1.00	6.00
	LOS average endpoint excursion (%)	70.58	13.71	36.63	61.25	74.50	80.00	104.60
	LOS movement velocity (m/s)	3.35	1.02	1.35	2.64	3.31	4.01	7.35
	LOS number of falls	0.43	0.77	0.00	0.00	0.00	1.00	3.00
	LOS number of falls on targets 4, 5, and 6	0.33	0.61	0.00	0.00	0.00	1.00	2.00
	LOS average endpoint excursion on targets 4, 5, and 6 (%)	61.99	16.45	26.33	49.67	61.84	72.00	103.33
	MCT average latency (ms)	144.12	9.21	118.00	139.00	145.50	150.00	161.00
	MCT average amplitude to large perturbations (degrees/second)	10.23	3.52	3.00	7.75	9.63	12.50	19.75
	MCT average posterior amplitude (degrees/second)	7.68	2.72	2.17	5.33	7.17	10.00	13.67
	MCT average posterior latency (ms)	151.98	10.84	125.00	145.00	151.67	161.67	170.00

Healthy cohort (*N*=55)	SOT composite equilibrium (1–100)	74.35	8.05	48.00	70.00	75.00	80.00	86.00
	SOT vestibular ratio (0-1)	0.64	0.16	0.00	0.57	0.67	0.75	0.92
	SOT visual preference	0.99	0.11	0.72	0.93	1.00	1.05	1.42
	SOT fall number	0.36	0.80	0.00	0.00	0.00	0.00	4.00
	SOT fall number-conditions 5 and 6	0.35	0.80	0.00	0.00	0.00	0.00	4.00
	LOS average endpoint excursion (%)	72.97	14.19	36.00	64.13	75.88	83.50	97.75
	LOS movement velocity (m/s)	4.45	1.45	2.29	3.26	4.18	5.79	8.06
	LOS number of falls	0.40	0.89	0.00	0.00	0.00	0.00	4.00
	LOS number of falls on targets 4, 5, and 6	0.16	0.57	0.00	0.00	0.00	0.00	3.00
	LOS average endpoint excursion on targets 4, 5, and 6 (%)	62.42	16.57	14.67	50.00	62.33	74.67	97.67
	MCT average latency (ms)	142.62	8.77	126.00	136.00	141.00	148.00	165.00
	MCT average amplitude to large perturbations (degrees/second)	10.19	2.82	5.50	7.75	10.00	11.75	16.50
	MCT average posterior amplitude (degrees/second)	7.18	2.22	3.50	5.50	7.00	8.17	12.50
	MCT average posterior latency (ms)	149.91	11.15	131.67	141.67	146.67	158.33	185.00

SOT, sensory organization test; LOS, limits of stability test; MCT, motor control test; SD, standard deviation; Q1, lower quartile; Q3, upper quartile.

**Table 4:** Discriminative validity of force platform measures between PD and healthy cohorts.

Force platform variable	*T* statistic (df)	*P* value	95% confidence interval
SOT composite equilibrium (1–100)	2.56 (64.563)	0.013^*∗*^	1.286, 10.357
SOT vestibular ratio (0-1)	2.26 (68.555)	0.027^*∗*^	0.012, 0.185
SOT visual preference	0.37 (80.477)	0.710	−0.039, 0.057
SOT fall number	−2.53 (50.466)	0.015^*∗*^	−1.527, −0.175
LOS average endpoint excursion (%)	0.81 (89.681)	0.422	−3.367, 7.964
LOS movement velocity (m/s)	4.36 (94.456)	<0.001^*∗*^	0.597, 1.595
LOS number of falls	−0.17 (93.594)	0.866	−0.365, 0.308
LOS number of falls on targets 4, 5, and 6	−1.39 (85.006)	0.167	−0.412, 0.072
LOS average endpoint excursion on targets 4, 5, and 6 (%)	0.13 (88.294)	0.899	−6.288, 7.512
MCT average latency (ms)	−0.81 (86.129)	0.419	−5.175, 2.174
MCT average amplitude to large perturbations (degrees/second)	−0.06 (76.892)	0.949	−1.364, 1.279

Independent *t*-tests between Parkinson's and healthy cohort are represented. ^*∗*^The significance level set at alpha <0.05. SOT, sensory organization test; LOS, limits of stability test; MCT, motor control test; df, degrees of freedom.

## Data Availability

The quantitative data on the force platform variables in the PD and healthy cohorts and statistical data used to support the findings of this study are included within the article.

## References

[1] DeMaagd G., Philip A. (2015). Parkinson’s disease and its management: part 1: disease entity, risk factors, pathophysiology, clinical presentation, and diagnosis. *Pharmacy and Therapeutics*.

[2] Warren Olanow C., Schapira A. H. V., Hauser S. L., Jospehson S. A. (2013). Parkinson’s disease and other extrapyramidal movement disorders. *Harrison’s Neurology in Clinical Medicine*.

[3] Kowal S. L., Dall T. M., Chakrabarti R., Storm M. V., Jain A. (2013). The current and projected economic burden of Parkinson’s disease in the United States. *Movement Disorders*.

[4] Allen N. E., Schwarzel A. K., Canning C. G. (2013). Recurrent falls in Parkinson’s disease: a systematic review. *Parkinson’s Disease*.

[5] Bloem B. R., Grimbergen Y. A., Cramer M., Willemsen M., Zwinderman A. H. (2001). Prospective assessment of falls in Parkinson’s disease. *Journal of Neurology*.

[6] Michatowska M., Fiszer U., Krygowska-Wajs A., Owczarek K. (2005). Falls in Parkinson’s disease. Causes and impact on patients’ quality of life. *Functional Neurology*.

[7] Rossi M., Soto A., Santos S., Sesar A., Labella T. (2009). A prospective study of alterations in balance among patients with Parkinson’s disease. Protocol of the postural evaluation. *European Neurology*.

[8] Mak M. K. Y., Pang M. Y. C. (2009). Balance confidence and functional mobility are independently associated with falls in people with Parkinson’s disease. *Journal of Neurology*.

[9] Matinolli M., Korpelainen J. T., Sotaniemi K. A., Myllylä V. V., Korpelainen R. (2011). Recurrent falls and mortality in Parkinson’s disease: a prospective two-year follow-up study. *Acta Neurologica Scandinavica*.

[10] Kim S. D., Allen N. E., Canning C. G., Fung V. S. C. (2013). Postural instability in patients with Parkinson’s disease. Epidemiology, pathophysiology and management. *CNS Drugs*.

[11] Kerr G. K., Worringham C. J., Cole M. H., Lacherez P. F., Wood J. M., Silburn P. A. (2010). Predictors of future falls in Parkinson disease. *Neurology*.

[12] Morris M. E., Iansek R. (1997). Gait disorders in Parkinson’s disease: a framework for physical therapy practice. *Neurology Report*.

[13] Bloem B. R. (1992). Postural instability in Parkinson’s disease. *Clinical Neurology and Neurosurgery*.

[14] Frank J. S., Horak F. B., Nutt J. (2000). Centrally initiated postural adjustments in parkinsonian patients on and off levodopa. *Journal of Neurophysiology*.

[15] Huh Y. E., Hwang S., Kim K., Chung W.-H., Youn J., Cho J. W. (2016). Postural sensory correlates of freezing of gait in Parkinson’s disease. *Parkinsonism & Related Disorders*.

[16] Robinson K., Dennison A., Roalf D. (2005). Falling risk factors in Parkinson’s disease. *NeuroRehabilitation*.

[17] Bloem B. R., Marinus J., Almeida Q. (2016). Measurement instruments to assess posture, gait, and balance in Parkinson’s disease: critique and recommendations. *Movement Disorders*.

[18] (2016). *PDEDGE Summary Documents*.

[19] Godi M., Franchignoni F., Caligari M., Giordano A., Turcato A. M., Nardone A. (2013). Comparison of reliability, validity, and responsiveness of the mini-BESTest and berg balance scale in patients with balance disorders. *Physical Therapy*.

[20] Leddy A. L., Crowner B. E., Earhart G. M. (2011). Utility of the mini-BESTest, BESTest, and BESTest sections for balance assessments in individuals with Parkinson disease. *Journal of Neurologic Physical Therapy*.

[21] King L. A., Salarian A., Mancini M. (2013). Exploring outcome measures for exercise intervention in people with Parkinson’s disease. *Parkinson’s Disease*.

[22] Nonnekes J., de Kam D., Guerts A. C., Weerdesteyn V., Bloem B. R. (2013). Unraveling the mechanism underlying postural instability in Parkinsons disease using dynamic posturography. *Expert Review of Neurotherapeutics*.

[23] Wallmann H. W. (2001). Comparison of elderly nonfallers and fallers on performance measures of functional reach, sensory organization, and limits of stability. *Journals of Gerontology Series A: Biological Sciences and Medical Sciences*.

[24] Girardi M., Konrad H. R., Amin M., Hughes L. F. (2001). Predicting fall risks in an elderly population: computer dynamic posturography versus electronystagmography test results. *Laryngoscope*.

[25] Whitney S. L., Marchetti G. F., Schade A. I. (2006). The relationship between falls history and computerized dynamic posturography in persons with balance and vestibular disorders. *Archives of Physical Medicine and Rehabilitation*.

[26] Faraldo-García A., Santos-Pérez S., Crujeiras R., Soto-Varela A. (2016). Postural changes associated with ageing on the sensory organization test and the limits of stability in healthy subjects. *Auris Nasus Larynx*.

[27] Chien C.-W., Hu M.-H., Tang P.-F., Sheu C.-F., Hsieh C.-L. (2007). A comparison of psychometric properties of the smart balance master system and the postural assessment scale for stroke in people who have had mild stroke. *Archives of Physical Medicine and Rehabilitation*.

[28] Liston R. A. L., Brouwer B. J. (1996). Reliability and validity of measures obtained from stroke patients using the balance master. *Archives of Physical Medicine and Rehabilitation*.

[29] Newstead A. H., Hinman M. R., Tomberlin J. A. (2005). Reliability of the berg balance scale and balance master limits of stability tests for individuals with brain injury. *Journal of Neurologic Physical Therapy*.

[30] Molina-Rueda F., Molero-Sánchez A., Alguacil-Diego I. M., Carratalá-Tejada M., Cuesta-Gómez A., Miangolarra-Page J. C. (2016). Weight symmetry and latency scores for unexpected surface perturbations in subjects with traumatic and vascular unilateral transtibial amputation. *PM&R*.

[31] Harro C. C., Marquis A., Piper N., Burdis C. (2016). Reliability and validity of force platform measures of balance impairment in individuals with Parkinson disease. *Physical Therapy*.

[32] Boonstra T. A., van Kordelaar J., Engelhart D., van Vugt J. P. P., van der Kooij H. (2016). Asymmetries in reactive and anticipatory balance control are of similar magnitude in Parkinson’s disease patients. *Gait & Posture*.

[33] Błaszczyk J. W. (2016). The use of force-plate posturography in the assessment of postural instability. *Gait & Posture*.

[34] Ferrazzoli D., Fasano A., Maestri R. (2015). Balance dysfunction in Parkinson’s disease: the role of posturography in developing a rehabilitation program. *Parkinson’s Disease*.

[35] Lahr J., Pinto Pereira M., Silva Pelicioni P. H., De Morais L. C., Bucken Gobbi L. T. (2015). Parkinson’s disease patients with dominant hemibody affected by the disease rely more on vision to maintain upright postural control. *Perceptual and Motor Skills*.

[36] Rossi-Izquierdo M., Basta D., Rubio-Rodríguez J. P. (2014). Is posturography able to identify fallers in patients with Parkinson’s disease?. *Gait & Posture*.

[37] Colnat-Coulbois S., Gauchard G. C., Maillard L. (2011). Management of postural sensory conflict and dynamic balance control in late-stage Parkinson’s disease. *Neuroscience*.

[38] Suarez H., Geisinger D., Suarez A., Carrera X., Buzo R., Amorin I. (2009). Postural control and sensory perception in patients with Parkinson’s disease. *Acta Oto-Laryngologica*.

[39] Doná F., Aquino C. C., Gazzola J. M. (2015). Changes in postural control in patients with Parkinson’s disease: a posturographic study. *Physiotherapy*.

[40] Yang Y.-R., Lee Y.-Y., Cheng S.-J., Lin P.-Y., Wang R.-Y. (2008). Relationships between gait and dynamic balance in early Parkinson’s disease. *Gait & Posture*.

[41] Frenklach A., Louie S., Koop M. M., Bronte-Stewart H. (2009). Excessive postural sway and the risk of falls at different stages of Parkinson’s disease. *Movement Disorders*.

[42] Dimitrova D., Horak F. B., Nutt J. G. (2004). Postural muscle responses to multidirectional translations in patients with Parkinson’s disease. *Journal of Neurophysiology*.

[43] Horak F. B., Dimitrova D., Nutt J. G. (2005). Direction-specific postural instability in subjects with Parkinson’s disease. *Experimental Neurology*.

[44] Lee J. M., Koh S.-B., Chae S. W. (2012). Postural instability and cognitive dysfunction in early Parkinson’s disease. *Canadian Journal of Neurological Sciences*.

[45] Liao Y.-Y., Yang Y.-R., Cheng S.-J., Wu Y.-R., Fuh J.-L., Wang R.-Y. (2015). Virtual reality–based training to improve obstacle-crossing performance and dynamic balance in patients with Parkinson’s disease. *Neurorehabilitation and Neural Repair*.

[46] Harro C., Shoemaker M. J., Frey O. J. (2014). The effects of speed-dependent treadmill training and rhythmic auditory-cued overground walking on balance function, fall incidence, and quality of life in individuals with idiopathic Parkinson’s disease: a randomized controlled trial. *NeuroRehabilitation*.

[47] Dalrymple-Alford J. C., MacAskill M. R., Nakas C. T. (2010). The MoCA: well-suited screen for cognitive impairment in Parkinson disease. *Neurology*.

[48] Perkins B., Olaleye D., Zinman B., Bril V. (2001). Simple screening tests for peripheral neuropathy in the diabetes clinic. *Diabetes Care*.

[49] Stebbins G. T., Goetz C. G., Burn D. J., Jankovic J., Khoo T. K., Tilley B. C. (2013). How to identify tremor dominant and postural instability/gait difficulty groups with the movement disorder society unified Parkinson’s disease rating scale: comparison with the unified Parkinson’s disease rating scale. *Movement Disorders*.

[50] Faul F., Erdfelder E., Lang A.-G., Buchner A. (2007). G∗Power 3: a flexible statistical power analysis program for the social, behavioral, and biomedical sciences. *Behavior Research Methods*.

[51] Portney L. G., Watkins M. P. (2009). *Foundations of Clinical Research: Applications to Practice*.

[52] LaPierre T. A., Hughes M., Uhlenberg P. (2009). Demography of aging in Canada and the United States. *International Handbook of Population Aging*.

[53] Ganesan M., Pal P. K., Gupta A., Sathyaprabha T. N. (2010). Dynamic posturography in evaluation of balance in patients of Parkinson’s disease with normal pull test: concept of a diagonal pull test. *Parkinsonism & Related Disorders*.

[54] Yelnik A. P., Le Breton F., Colle F. M. (2008). Rehabilitation of balance after stroke with multisensorial training: a single-blind randomized controlled study. *Neurorehabilitation and Neural Repair*.

[55] Nematollahi A., Kamali F., Ghanbari A., Etminan Z., Sobhani S. (2016). Improving balance in older people: a double-blind randomized clinical trial of three modes of balance training. *Journal of Aging and Physical Activity*.

[56] Sparrow D., DeAngelis T. R., Hendron K., Thomas C. A., Saint-Hilaire M., Ellis T. (2016). Highly challenging balance program reduces fall rate in Parkinson disease. *Journal of Neurologic Physical Therapy*.

